# Hybrid *Cannabis sativa* L. inflorescences exert an anti-inflammatory effect through the modulation of MAPK/NF-κB/NLRP3 inflammasome and JAK1/STAT6 pathway in HaCaT cells

**DOI:** 10.3389/fphar.2025.1617180

**Published:** 2025-07-24

**Authors:** Ji-Ye Han, Do-Won Lim, Osoung Kwon, Yun Jung Lee, Hye Yung Choi, Hyun-Ju Jung, Soohyang Noh, Mansoo Cho, Sung Suk Kang, Young-Mi Lee

**Affiliations:** ^1^ Department of Oriental Pharmacy, College of Pharmacy, Wonkwang University, Iksan, Republic of Korea; ^2^ Wonkwang-Oriental Medicines Research Institute, Wonkwang University, Iksan, Republic of Korea; ^3^ Graduate School of Techno Design, Kookmin University, Seoul, Republic of Korea; ^4^ Korea Dispensary Inc., Cheongju, Republic of Korea; ^5^ The One Health Design Inc., Iksan, Republic of Korea

**Keywords:** *Cannabis sativa* L., atopic dermatitis, inflammation, inflammatory diseases, skin

## Abstract

**Background:**

Atopic dermatitis (AD) is a chronic inflammatory skin disease accompanied by severe itching. Reducing mediators of skin inflammation and itching is crucial for the treatment of AD. *Cannabis sativa* L. contains many types of cannabinoids and flavonoids, which exhibit antioxidant and anti-inflammatory effects. This study aims to demonstrate the anti-inflammatory and anti-atopic dermatitis effects of hybrid *C. sativa* L. inflorescence extracts (HCIE) in human keratinocytes.

**Methods:**

*Cannabis sativa* extracts were analyzed using UPLC. Gene expression levels in HCIE-treated HaCaT cells were measured by RT-PCR, and intracellular ROS were evaluated using DCF-DA. Protein expression levels related to MAPK, NF-κB, NLRP3 inflammasome, and JAK1/STAT6 pathways were determined by immunoblotting.

**Results:**

The UPLC analysis revealed that a total of 8 cannabinoids were detected in HCIE. Among the cannabinoids identified in HCIE, CBDA and CBD were the most abundant, collectively accounting for approximately 28% of the total extract. The gene expression of MDC, RANTES, and TARC exhibited dose-dependent suppression in the HCIE-treated group. MAPK phosphorylation was inhibited in the HCIE-treated group. Additionally, NF-kB, p-NF-kB, NLRP3, and caspase-1 were reduced in a dose-dependent manner by HCIE. The activation of JAK1 and STAT6 was diminished in HaCaT cells treated with HCIE. Conversely, the levels of filaggrin and involucrin were significantly elevated in the HCIE-treated group compared to the control group.

**Conclusion:**

Taken together, HCIE suppresses inflammation mediators through the regulation of the MAPK/NF-κB/NLRP3 inflammasome and JAK1/STAT6 pathways, while up-regulating skin moisturizing factors in keratinocytes. These results suggest that HCIE may be utilized in the treatment of skin inflammatory diseases, such as AD.

## 1 Introduction

Atopic dermatitis (AD) is a common skin disease in children. Excessive immune response due to an abnormality in the immune system causes severe itching, and a vicious cycle of itching and scratching occurs repeatedly (Wahlgren, 1999). This vicious cycle destroys the skin barrier and facilitates the penetration of external antigens (Yosipovitch et al., 2019). Penetrated antigens promote the immune response of the skin and cause inflammation (Proksch et al., 2009). The causes of AD are diverse, including genetic or environmental factors, immune system dysfunction, and skin barrier abnormalities, and are closely related to T cell activation (Akdis et al., 2000). The imbalance of Th1/2 leads to the development of AD (Orciani et al., 2017). In the acute phase, the Th2 response is dominant, and the Th1 response is dominant in the chronic phase (Gittler et al., 2012). Increased IL-4 and IL-13 in the acute phase inhibit the transcription of skin barrier proteins through the JAK1/STAT6 pathway (Jiang et al., 2000; Bao et al., 2012). This inhibition leads to skin barrier damage.

Collapse of the skin barrier is a major feature of AD (Proksch et al., 2009). Functional defects and genetic mutations of skin components constitute an unstable skin barrier, preventing normal skin functions from being performed (Proksch et al., 2008). The skin epidermis is divided into the basal layer, spinous layer, granular layer, and stratum corneum from the basement membrane; the granular layer is composed of keratinocytes and proteins such as filaggrin, loricrin, and involucrin (Rerknimitr et al., 2018). In particular, filaggrin is processed from profilaggrin to filaggrin in the granular layer, which produces natural moisturizing factors and maintains the acidity of the epidermis (Sandilands et al., 2009). In AD lesions, filaggrin is reduced, and the tight junctions between cells are weakened (Oh et al., 2021). This incomplete skin composition loosens the skin barrier, causing moisture loss and changing the skin into a dry environment (Kim and Lim, 2021).

Macrophage-derived chemokine (MDC), Regulated upon activation, T cell expressed and presumably secreted (RANTES), and Thymus-and activation-regulated chemokine (TARC) are key biomarkers of AD and are significantly up-regulated in AD (Renert-Yuval et al., 2021). These chemokines attract immune cells, including leukocytes, T cells, and mast cells, to the site of inflammation and activate Th1 and Th2 cells, thereby promoting the production of pro-inflammatory cytokines (Rapp et al., 2019). Furthermore, these molecules serve as key mediators of allergic and inflammatory diseases (Castan et al., 2017).

Currently, topical corticosteroids are predominantly used in the treatment of AD. Although these agents demonstrate potent anti-inflammatory effects owing to their immunosuppressive properties, they are accompanied by several side effects, including an increased risk of infection, skin atrophy, and hypertension (Stacey and McEleney, 2021).

Herbal medicine serves as a promising alternative to synthetic drugs, which are often associated with numerous side effects. *Cannabis sativa* L. is a plant that has been used by humans for a long time. It was first cultivated in Central Asia and is currently cultivated in a wide area, including Asia, Europe, and America. *C. sativa* has been used for herbal medicine as well as food and fiber, and is a good material for rope and fabric (Manaia et al., 2019).

*Cannabis* species contain various cannabinoids, among which Δ^9^-tetrahydrocannabinol (Δ^9^-THC) and cannabidiol (CBD) are the most well-known (Castillo-Arellano et al., 2023). *Cannabis sativa* contains approximately 110 different phytocannabinoids, including cannabichromene (CBC), cannabigerol (CBG), and cannabinol (CBN); terpenoids, flavonoids, and alkaloids have also been isolated from *C. sativa* (Gulck and Moller, 2020). According to previous studies, *C. sativa* exhibits the effect of antioxidant, anti-inflammatory, antibacterial, and anticancer (Kumar et al., 2021). In addition, although it exhibits strong analgesic properties and is primarily used as a pain reliever in clinical settings, its application is limited due to significant psychoactive side effects (van de Donk et al., 2019). Currently, several medications containing CBD and THC as the primary ingredients have been developed and are prescribed exclusively for the treatment of patients with epilepsy, multiple sclerosis, and those undergoing cancer treatment (Syed et al., 2014).

*Cannabis sativa* has also been investigated for its effects on skin diseases. Previous studies have demonstrated that *C. sativa* extracts or cannabinoids exert significant therapeutic effects in improving conditions such as skin inflammation, skin cancer, psoriasis, and AD (Martinelli et al., 2022; Wolińska et al., 2025). However, most studies on *C. sativa* have primarily focused on CBD and THC, with relatively limited studies exploring other cannabinoids, terpenoids, polyphenols, and extracts. Recent studies have reported the entourage effect of components contained in *C. sativa.* The entourage effect refers to the synergistic effect that occurs from the interaction of cannabinoids or cannabinoids and terpenoids (LaVigne et al., 2021). This suggests that compounds other than CBD and THC present in *C. sativa* play a significant role in its diverse effects, meaning the need for further research into their roles and interactions. Additionally, this implies that research on complex compounds, such as *C. sativa* extract, is as important as studies focusing on individual compounds like CBD and THC.

Hybrid *C. sativa* is a variety developed by crossbreeding Korean native species with foreign species. This study investigated the inhibitory effect of hybrid *C. sativa* ethanol extract on skin inflammation. Furthermore, the potential of hybrid *C. sativa* ethanol extract to improve AD through the modulation of AD mediators and skin moisturizing factors was explored *in vitro*.

## 2 Materials and methods

### 2.1 Preparation of hybrid *Cannabis sativa* L. Inflorescence extracts (HCIE)

A hybrid *C. sativa* strain (IT342820) deposited at the National Agrobiodiversity Center (RDA, Korea) was used in this study. Hybrid *C. sativa* inflorescences were provided by Nongboomind (Andong, Korea). Dried hybrid *C. sativa* inflorescences 10 g were soaked in 70% ethanol 100 mL and subjected to ultrasonic extraction (40 kHz, 40°C–50°C). This process was repeated three times for 30 min each. The solution was filtered through a 6 μm filter paper (ADVANTEC, Tokyo, Japan) and concentrated under reduced pressure with a rotary vacuum evaporator at 40°C (EYELA N-1110, Tokyo, Japan). HCIE powder was obtained by lyophilization at −80°C, and the final yield was 11.76%. HCIE was stored at −20°C until use. HCIE was dissolved in DMSO for treatment with cells, and the final concentration of DMSO used for cells was less than 0.1%.

### 2.2 Ultra-performance liquid chromatography analysis of hybrid *Cannabis sativa* L. Inflorescence extracts (HCIE)

Cannabinoid standards were provided by Korea Dispensary Inc (Cheongju, Korea). CBD was isolated and provided by Dr. Jung from the College of Pharmacy, Wonkwang University (Iksan, Korea). The standards used for analysis were diluted in acetonitrile, and an HCIE 1 mg/mL was prepared using 70% ethanol. In this analysis, cannabinoid single standards and mixtures were used, and the information on the standards used is shown in Supplementary Table S1. The prepared standards and HCIE were filtered using a 0.2 μm polytetrafluoroethylene syringe filter (Ø13 mm, Hyundai micro, Seoul, Korea). HCIE was analyzed using ultra-performance liquid chromatography (ACQUITY UPLC H-Class, Waters, Milford, MA, United States), and the column used was an ACQUITY UPLC BEH C18 Column, 1.7 µm, 2.1 mm × 100 mm (Waters, Milford, MA, United States) with an ACQUITY UPLC BEH C18 VanGuard Pre-column, 1.7 µm, 2.1 mm × 5 mm (Waters, Milford, MA, United States). The mobile phases consisted of (A) 0.1% formic acid in water and (B) 0.1% formic acid in acetonitrile and methanol (25:75, v/v). The gradient program was set as follows: 0–5 min 73.5%–77% B, 5–9 min 77%–90% B, 9–11 min 90% B, 11–15 min 90%–73.5% B. During the analysis, the column temperature was maintained at 30°C, and the mobile phase flow rate was 0.35 mL/min. The injection volume was 1.5 μL, and the detection wavelength of the extract was 220 nm.

### 2.3 Determination of total flavonoid content

The total flavonoid content (TFC) of the extract was measured using a slightly modified aluminum chloride colorimetry method (Do et al., 2014). Briefly, the extract was diluted to 1 mg/mL with ethanol. Quercetin was used as a standard, and a calibration curve was prepared with 0–100 μg/mL diluted quercetin in ethanol. Initially, 0.5 mL of samples were mixed with 10% (w/v) aluminum chloride, and 0.1 mL of 0.1 mM potassium acetate was added. Then, 2.8 mL of distilled water was added to adjust the volume of the mixture. The solution was reacted at room temperature for 30 min. After transferring 200 μL of each sample to a microplate, the absorbance was measured at 415 nm using a microplate reader (SpectraMax 190, Molecular Devices, San Jose, CA, United States). The flavonoid content was expressed as milligrams of quercetin equivalents per Gram of extract (mg QC/g).

### 2.4 Determination of total phenolic content

The total phenolic content (TPC) of the extract was measured using the Folin-Ciocalteu method (Ainsworth and Gillespie, 2007). Briefly, the extract and standard were diluted in methanol, and 1 mg/mL of the extract was prepared. Gallic acid was used as a standard for analysis, and a calibration curve was prepared with 0–100 μg/mL gallic acid. First, 200 μL of 10% (v/v) Folin-Ciocalteu reagent was added to 100 μL of each sample and standard. After mixing thoroughly, 800 μL of 700 mM sodium carbonate was added to each sample. The mixture was protected from light and reacted at room temperature for 2 h. Only 200 μL of each sample was transferred to a microplate, and the absorbance was measured at 765 nm using a microplate reader (SpectraMax 190, Molecular Devices, San Jose, CA, United States). The phenolic content was expressed as milligrams of gallic acid equivalents per Gram of extract (mg GA/g).

### 2.5 DPPH radical scavenging assay

The DPPH radical scavenging activity of HCIE was measured using a slightly modified method described by Frezzini et al. (2019). Briefly, 100 μM DPPH was prepared in ethanol and filtered through a 0.2 μm filter. Ascorbic acid was used as a control to compare the antioxidant activity of HCIE, and 20 μg/mL of ascorbic acid was used. The 25, 50, 100, and 200 μg/mL of HCIE were dissolved in ethanol, and the samples were reacted with DPPH at 37°C for 30 min. After that, the absorbance at 517 nm was measured using a microplate reader (SpectraMax 190, Molecular Devices, San Jose, CA, United States). The calculation formula for antioxidant activity is as follows:

$$\text{DPPH }\text{radical }\text{scavenging }\left(\%\right)=\left[\left(\text{Ac}-\text{As}\right)÷\text{Ac}\right]\times 100$$

where Ac is the absorbance of the control, and As is the absorbance of the sample.

### 2.6 Cell culture

The human keratinocyte HaCaT cell line was purchased from Cell Lines Service (Eppelheim, Germany). The cells were cultured in Dulbecco’s Modified Eagle’s Medium (DMEM) containing 10% fetal bovine serum (Thermo Fisher Scientific, Cleveland, OH, United States), 10 mM HEPES (WelGENE, Daegu, Korea), and 1% penicillin (1 × 10^4^ units/mL)-streptomycin (1 × 10^4^ μg/mL) (WelGENE, Daegu, Korea). The cells were incubated under the conditions of 5% CO_2_ at 37°C throughout the experiment.

### 2.7 Cell cytotoxicity assay

The cytotoxicity of HCIE on HaCaT cells was evaluated using the MTT assay (Plumb, 2004). MTT powder (Sigma-Aldrich, St. Louis, MO, United States) was dissolved in water to make a 5 mg/mL MTT solution, which was filtered using a 0.2 μm syringe filter. This solution was diluted to 50 μg/mL with DMEM and used for cells. HaCaT cells were seeded in 96-well plates at 1 × 10^4^ cells/well and cultured overnight. The cells were exposed to HCIE at 2.5, 5, 10, 20, and 40 μg/mL for 24 h. After the removal of cell supernatant, MTT was added to each well and reacted for 4 h. Subsequently, DMSO was added to dissolve the produced formazan crystals, and the absorbance was measured at 540 nm using a SpectraMAX 190 microplate reader (Molecular Devices, San Jose, CA, United States).

### 2.8 Real-time quantitative polymerase chain reaction (RT-PCR)

The total RNA of HaCaT cells was purified using the TRIzol Reagent method described by Rio et al. (2010). The isolated RNA was dissolved in RNase-free water and quantified using a BioSpectrometer (Eppendorf, Hamburg, Germany). cDNA synthesis was performed using the HelixCript Easy cDNA Synthesis kit (NanoHelix, Daejeon, Korea) according to the manufacturer’s protocol. A SimpliAmp Thermal Cycler (Applied Biosystems, Foster City, CA, United States) was used for cDNA synthesis. RT-PCR was performed on a StepOnePlus Real-Time PCR System (Applied Biosystems, Foster City, CA, United States), and a RealHelix Premier qPCR kit (NanoHelix, Daejeon, Korea) was used for this process. The primers used for gene amplification are shown in Supplementary Table S2, and the expression level of mRNA was normalized to GAPDH.

### 2.9 Enzyme-linked immunosorbent assay (ELISA)

The cells were pre-treated with HCIE (25, 50, 100, and 200 ng/mL) for 30 min and exposed to TNF-α/IFN-γ for 24 h. Cell culture medium was harvested and centrifuged at 200 × g for 5 min. Only the supernatant was collected and stored at −20°C until use. For the measurement of secreted cytokines, the RANTES ELISA kit (#DY278-05; R&D Systems, Minneapolis, MN, United States) and TARC ELISA kit (#DY364-05; R&D Systems, Minneapolis, MN, United States) were used. The protein levels of RANTES and TARC were determined according to the manufacturer’s guidelines. Absorbance was measured and quantified using a microplate reader (SpectraMax 190, Molecular Devices, San Jose, CA, United States).

### 2.10 Cell fractionation

HaCaT cells were fractionated using the NE-PER Nuclear and Cytoplasmic Extraction Kit (Thermo Fisher Scientific, Cleveland, OH, United States). Briefly, the cells were collected with culture medium, centrifuged, and the supernatant was discarded. The cell pellet was then prepared for fractionation, which was carried out according to the manufacturer’s protocol. The resulting cellular fractions were quantified using the Bradford reagent (Bio-Rad, Hercules, CA, United States) and stored at −20°C until use. Proteins contained in the fractions were detected by immunoblotting.

### 2.11 Immunoblotting

The proteins were extracted from whole-cell lysates using radioimmunoprecipitation assay buffer (ELPIS-Bioech, Daejeon, Korea) containing a Halt™ protease inhibitor cocktail (Thermo Fisher Scientific, Cleveland, OH, United States). Bradford reagent (Bio-Rad, Hercules, CA, United States) was used to quantify the extracted proteins, and the absorbance of the sample at 595 nm was measured using a microplate reader (SpectraMax 190, Molecular Devices, San Jose, CA, United States). SDS-polyacrylamide gel electrophoresis was performed for protein separation, and a 10% polyacrylamide gel was used. The separated proteins were transferred to a polyvinylidene fluoride membrane, and the membranes were blocked with 5% skim milk (w/v) for 1 h at room temperature. Then, the membranes were incubated with primary antibodies overnight at 4°C. All primary antibodies were diluted according to the manufacturer’s recommended concentration, and detailed information on the primary antibodies is shown in Supplementary Table S3. Horseradish peroxidase-conjugated secondary antibodies were reacted at room temperature for 1 h, and the proteins were detected by enhanced chemiluminescence reagent (Kindle Biosciences, Greenwich, CT, United States) using the ChemiDoc imaging system (Bio-Rad, Hercules, CA, United States).

### 2.12 Measurement of intracellular reactive oxygen species (ROS)

Intracellular ROS levels were assessed using 2′,7′-dichlorodihydrofluorescein diacetate (DCF-DA). The 10 μM DCF-DA was prepared by dissolving it in PBS. The cells were seeded in a 24-well plate (5 × 10^4^ cells/well) and incubated overnight. The cells were pre-treated with 25, 50, 100, 200 ng/mL, and 10 mM N-Acetyl-L-cysteine (NAC) for 30 min. Then, the cells were stimulated with 10 ng/mL each of TNF-α and IFN-γ for 30 min. After washing three times with ice-cold PBS, 10 μM DCF-DA was added and incubated at 37°C for 20 min in the dark. Washing was repeated and incubated with the cell culture medium for 10 min at 37°C. The intracellular ROS levels were analyzed by EVOS FLoid Color Imaging Systems (Thermo Fisher Scientific, Cleveland, OH, United States). The fluorescence intensity was measured using ImageJ software v1.54 (National Institutes of Health, Bethesda, MD, United States).

### 2.13 Statistical analysis

All data are shown as mean ± standard deviation (SD), and all the experiments were repeated at least three times. The differences between the groups were assessed using one-way analysis of variance (ANOVA) and followed by Tukey’s multiple comparison test. The values of p < 0.05 were considered statistically significant, and p-values were given as follows: *p < 0.05, **p < 0.01, ***p < 0.001. All analyses were performed using GraphPad Prism version 8.0 (GraphPad Software, San Diego, CA, United States).

## 3 Results

### 3.1 Ultra-Performance Liquid Chromatography Analysis of HCIE

**FIGURE 1:**
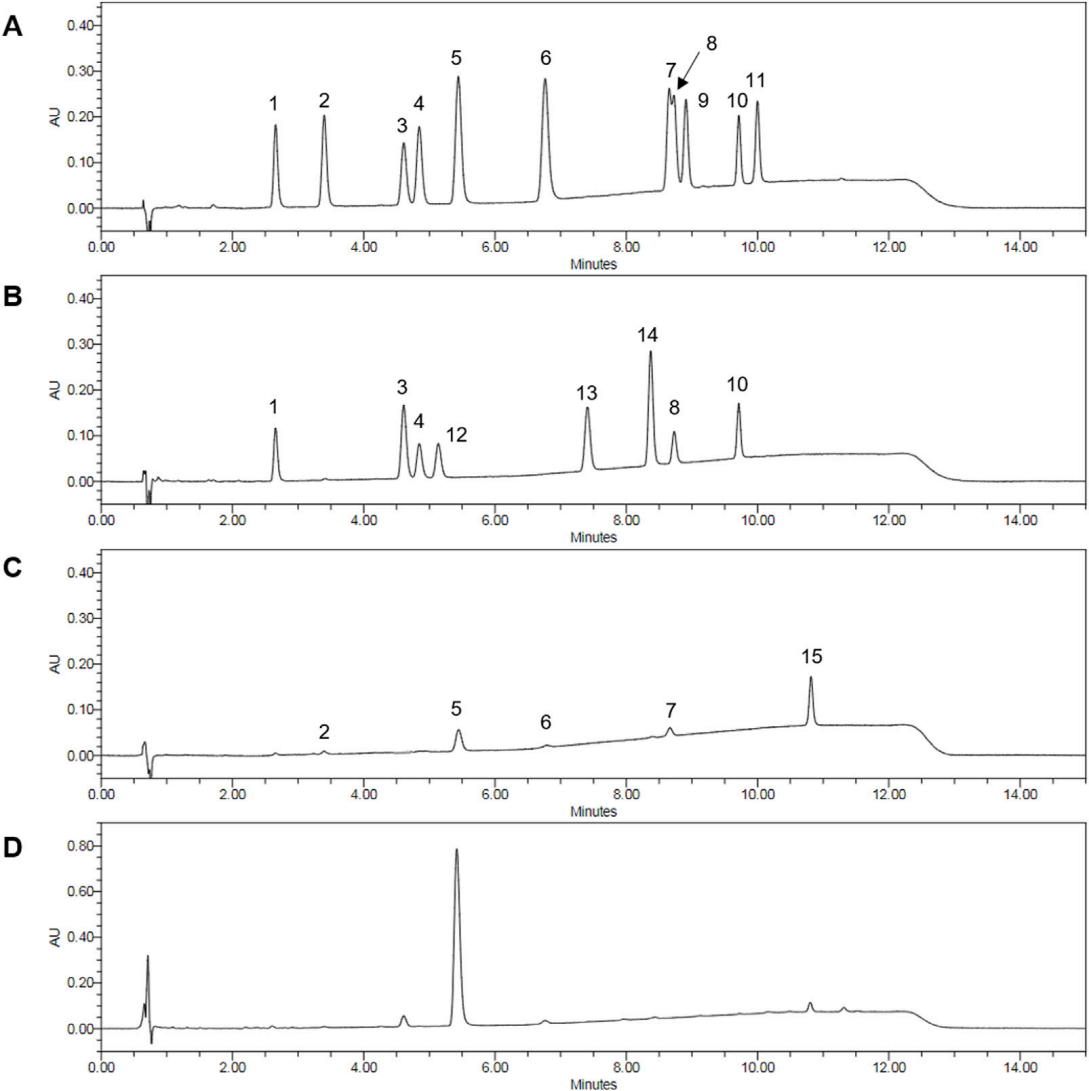
Ultra-Performance Liquid Chromatography Analysis of cannabinoid in HCIE. **(A)** Chromatogram of a cannabinoid single-compound standard mixture. **(B)** Chromatogram of cannabinoid neutral form standard mixture. **(C)** Chromatogram of cannabinoid acidic form standard mixture. **(D)** Chromatogram of HCIE. The peaks are represented as follows: 1 cannabidivarin (CBDV), 2 cannabidivarinic acid (CBDVA), 3 cannabidiol (CBD), 4 cannabigerol (CBG), 5 cannabidiolic acid (CBDA), 6 cannabigerolic acid (CBGA), 7 tetrahydrocannabivarinic acid (THCVA), 8 Δ^8^-tetrahydrocannabinol (Δ^8^-THC), 9 cannabicyclol (CBL), 10 cannabichromene (CBC), 11 cannabinolic acid (CBNA), 12 tetrahydrocannabivarin (THCV), 13 cannabinol (CBN), 14 Δ^9^-tetrahydrocannabinol (Δ^9^-THC), 15 tetrahydrocannabinolic acid (THCA).

The components contained in HCIE were analyzed using ultra-performance liquid chromatography. As a result of the analysis, a total of 8 cannabinoids were detected in HCIE: cannabidivarin (CBDV), cannabidivarinic acid (CBDVA), cannabidiol (CBD), cannabidiolic acid (CBDA), cannabigerolic acid (CBGA), tetrahydrocannabinolic acid (THCA), cannabichromene (CBC), and Δ^9^-tetrahydrocannabinol (Δ^9^-THC) (Figure 1). CBDA showed the highest content in the extract, with 261.79 μg per mg, followed by CBD at 16.39 μg. Together, CBD and CBDA accounted for approximately 28% of the total extract (Table 1).

**TABLE 1:** Cannabinoid, total flavonoid, and total polyphenol content of HCIE.

Cannabinoid	Content (μg/mg)	RT (min)	M.W. (g/mol)
CBDV	2.87 ± 0.09	2.66	286.41
CBDVA	1.57 ± 0.12	3.40	330.42
CBD	16.39 ± 0.16	4.61	314.46
CBG	N.D.	4.85	316.48
THCV	N.D.	5.14	286.41
CBDA	261.79 ± 0.6	5.44	358.47
CBGA	5.68 ± 0.18	6.76	360.49
CBN	N.D.	7.41	310.43
Δ^9^-THC	3.97 ± 0.03	8.37	314.46
THCVA	N.D.	8.66	330.42
Δ^8^-THC	N.D.	8.73	314.46
CBL	N.D.	8.91	314.46
CBC	1.27 ± 0.21	9.71	314.46
CBNA	N.D.	10.00	354.44
THCA	6.51 ± 0.1	10.82	358.47

	TFC (mg QC/g)	TPC (mg GA/g)
Content	62.57 ± 3.23	107.05 ± 7.05	

N.D., not detected.

### 3.2 Analysis of total flavonoid and total polyphenol content of HCIE

Total flavonoid content (TFC) was determined based on quercetin, and the total polyphenol content (TPC) was quantified based on gallic acid. The TFC of HCIE was 62.57 mg per g of extract, constituting approximately 6.3% of the extract, while the TPC was 107.05 mg, representing approximately 11% of the extract (Table 1).

### 3.3 HCIE reduces gene expression of inflammatory mediators in human keratinocytes

**FIGURE 2:**
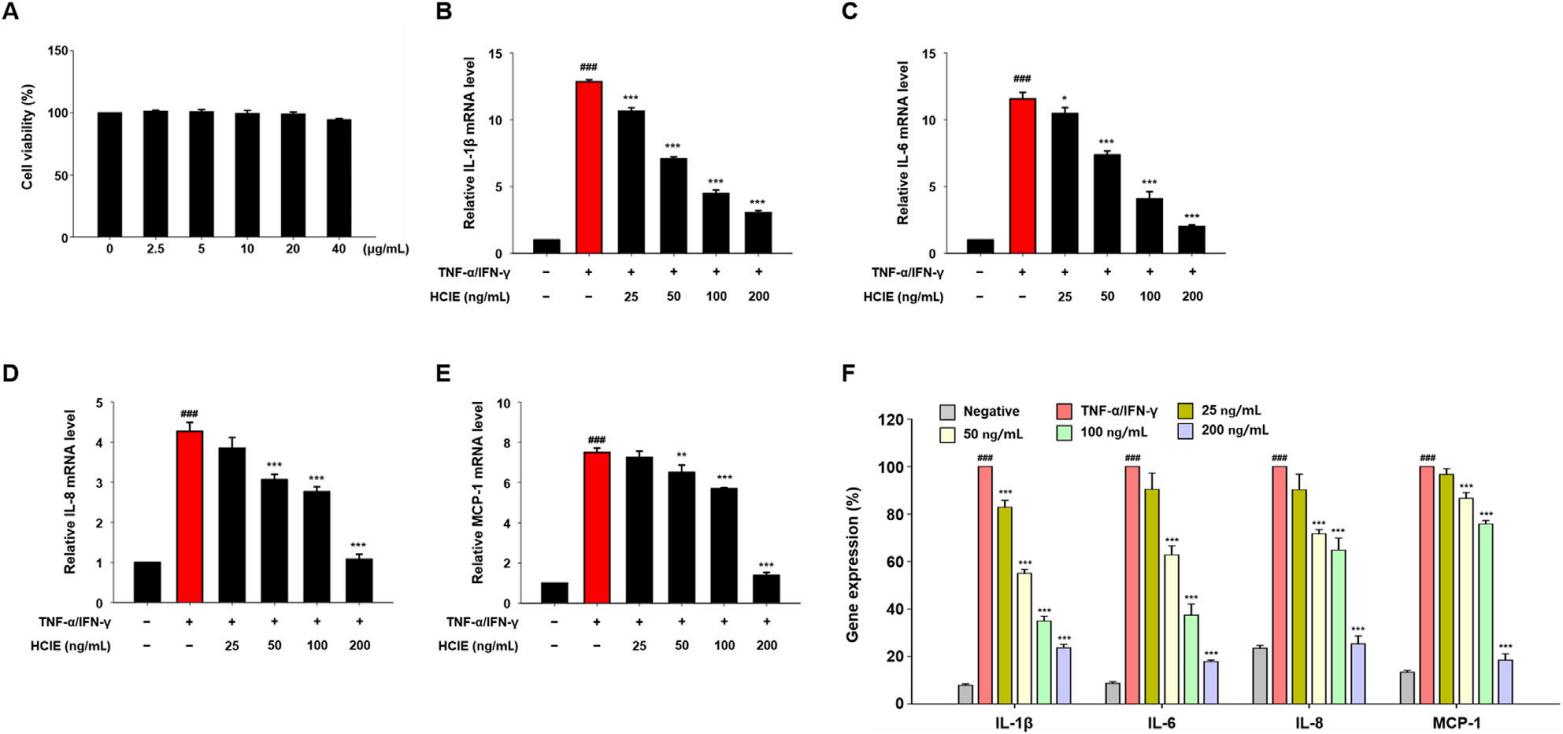
Inhibitory effect of HCIE on pro-inflammatory cytokines expression in HaCaT cells. The cytotoxicity of HCIE was evaluated using the MTT assay. The cells were co-cultured with HCIE at 2.5, 5, 10, 20, and 40 μg/mL for 24 h. **(A)** This graph shows the cytotoxicity of HCIE in HaCaT cells, and 0 μg/mL is the solvent control. Gene expression of pro-inflammatory cytokines was analyzed by RT-PCR. The cells were pre-treated with HCIE at 25, 50, 100, and 200 ng/mL for 30 min. Then, they were stimulated with TNF-α/IFN-γ (each 10 ng/mL) for 2 h. The mRNA level was normalized to GAPDH, and relative mRNA levels of **(B)** IL-1β, **(C)** IL-6, **(D)** IL-8, and **(E)** MCP-1 were measured. **(F)** The percentage of relative gene expression was calculated. Data represented as mean ± SD (*n* = 3) and the *p* values are presented on the graph as follows: ^###^ *p* < 0.001 vs. control group; *p < 0.05, **p < 0.01, and ***p < 0.001 vs TNF-α/IFN-γ induced group.

**FIGURE 3:**
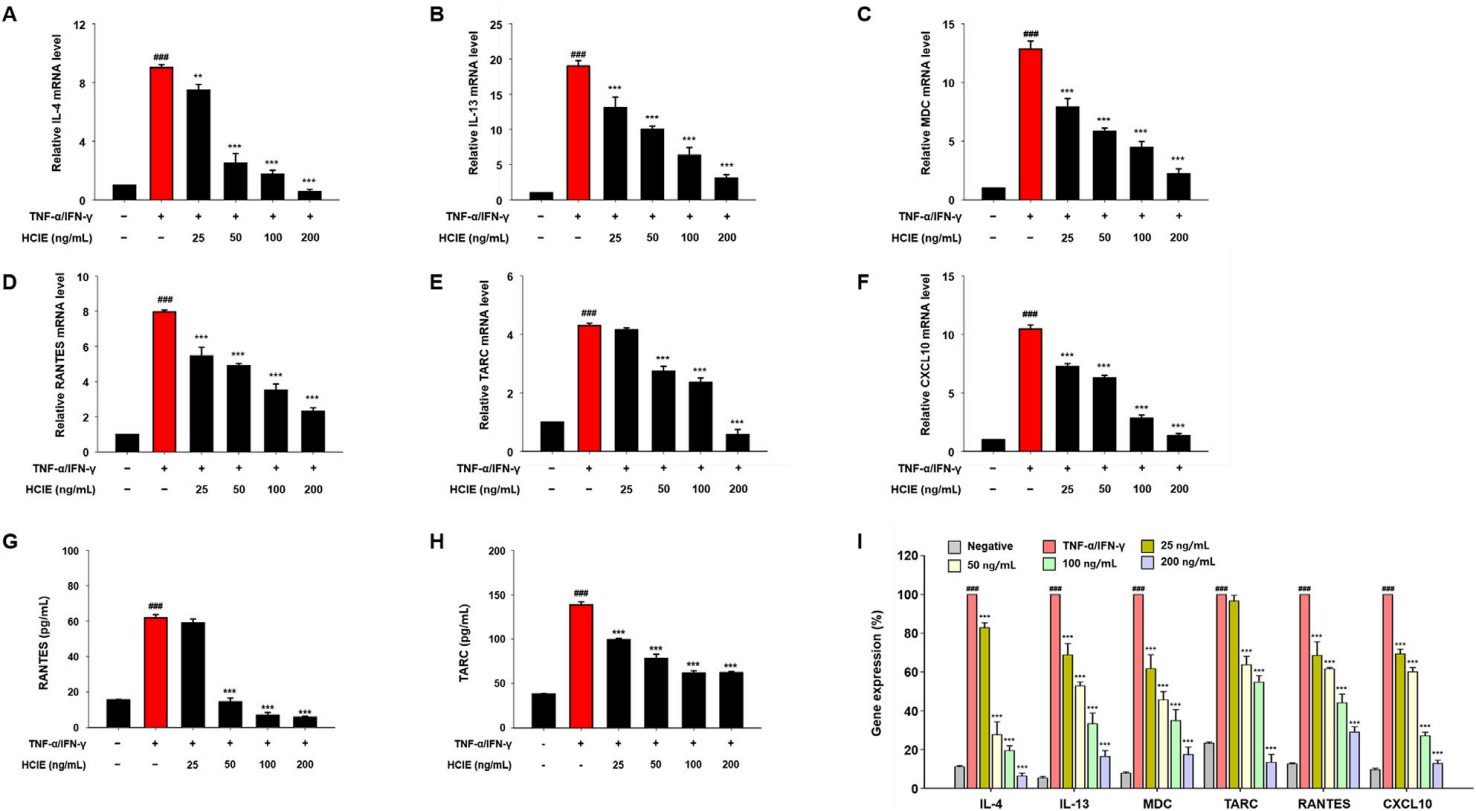
Inhibitory effect of HCIE on AD-related cytokines expression in HaCaT cells. Gene expression of AD-related cytokines was measured using RT-PCR. The cells were pre-treated with the indicated concentrations of HCIE for 30 min and stimulated with TNF-α/IFN-γ (each 10 ng/mL). The relative mRNA levels of **(A)** IL-4, **(B)** IL-13, **(C)** MDC, **(D)** RANTES, **(E)** TARC, and **(F)** CXCL10 were measured, and mRNA was normalized to GAPDH. Secreted **(G)** RANTES and **(H)** TARC were evaluated using ELISA. **(I)** The percentage of relative gene expression was calculated. Data represented as mean ± SD (*n* = 3) and the *p* values are presented on the graph as follows: ^###^ *p* < 0.001 vs control group; **p < 0.01, and ***p < 0.001 vs TNF-α/IFN-γ induced group.

Before treating cells with HCIE, the cytotoxicity of HCIE on HaCaT cells was evaluated. The cells were co-cultured with HCIE at 2.5, 5, 10, 20, and 40 μg/mL, and the cell viability remained above 90% at all concentrations. To investigate the effect of HCIE on inflammation, the mRNA expression levels of pro-inflammatory cytokines were measured in HCIE-treated cells. The mRNA expression levels of IL-1β, IL-6, IL-8, and MCP-1 were dose-dependently down-regulated following HCIE treatment. The mRNA expression of pro-inflammatory cytokines in the group treated with 200 ng/mL of HCIE was reduced by up to 82.17% compared to the group treated with TNF-α/IFN-γ (Figure 2). In addition, the mRNA expression levels of IL-4 and IL-13 were dose-dependently reduced in the HCIE-treated group. The mRNA expression levels of MDC, RANTES, TARC, and CXCL10 were also suppressed by HCIE (Figures 3A–F,I). Secretion of RANTES was significantly inhibited at concentrations of HCIE 50 ng/mL or higher, while secretion of TARC was inhibited even at low concentrations of HCIE (Figures 3G,H).

### 3.4 HCIE decreases intracellular ROS in human keratinocytes

**FIGURE 4:**
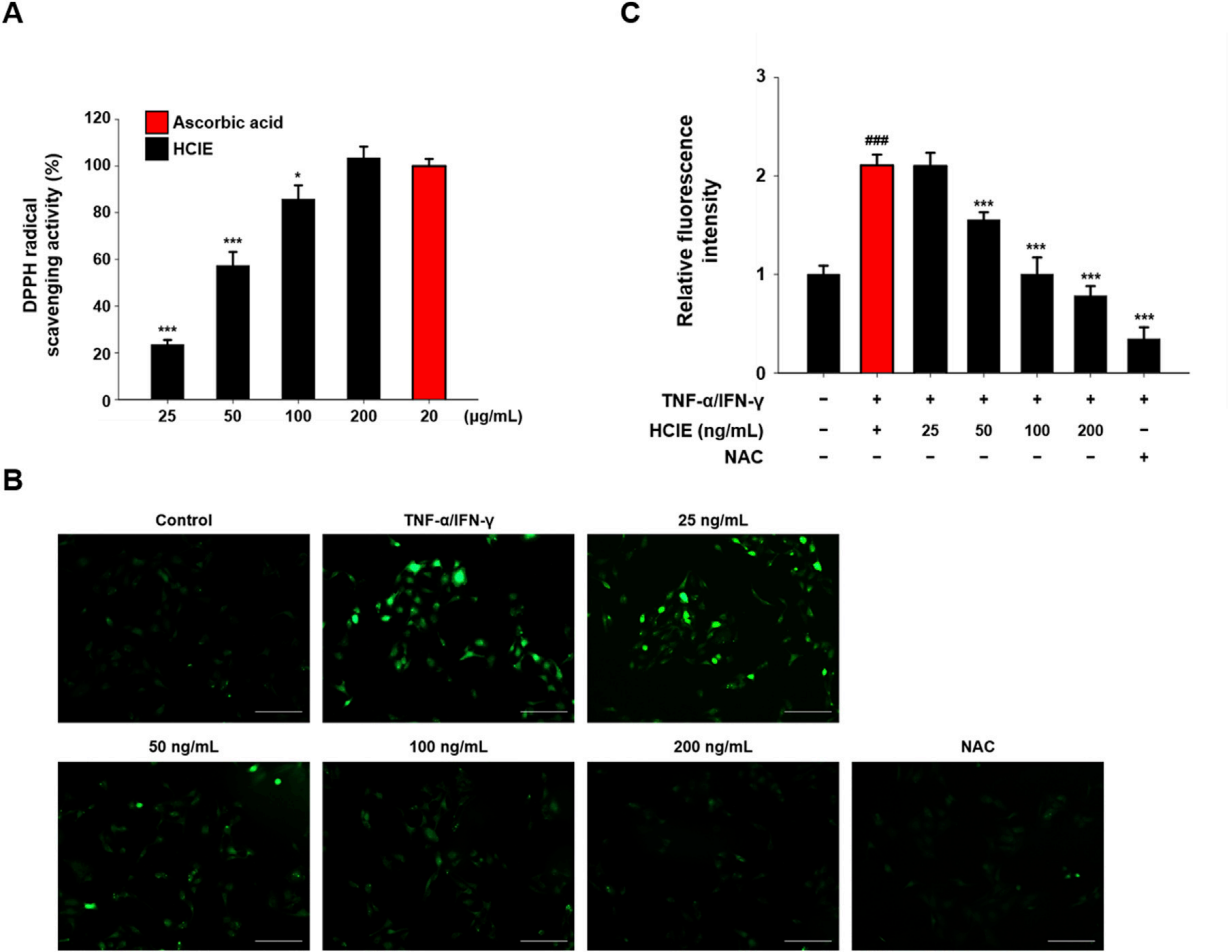
Antioxidant effect of HCIE in HaCaT cells. **(A)** DPPH radical scavenging activity of HCIE. Ascorbic acid was used as a control. HaCaT cells were pre-treated with HCIE (25, 50, 100, and 200 ng/mL) or NAC (10 mM) for 30 min and then stimulated with 10 ng/mL of TNF-α/IFN-γ. **(B)** Representative fluorescence microscopy images (×200 magnification, scale bar: 200 μm) of DCF-DA staining in HCIE-treated HaCaT cells. **(C)** Relative fluorescence intensity compared to the normal control. Data represented as mean ± SD (*n* = 3) and the *p* values are presented on the graph as follows: ^###^ *p* < 0.001 vs. control group; *p < 0.05 and ***p < 0.001 vs. TNF-α/IFN-γ induced group.

To investigate the antioxidant effect of HCIE, the DPPH radical scavenging activity and intracellular ROS levels of HCIE were measured in HCIE-treated HaCaT cells. The DPPH radical scavenging activity of HCIE increased in a dose-dependent manner, with the activity at 200 μg/mL of HCIE being comparable to that of 20 μg/mL of ascorbic acid (Figure 4A). The intracellular ROS levels were significantly increased in TNF-α/IFN-γ induced HaCaT cells, but were reduced by NAC. In HaCaT cells treated with 25 ng/mL HCIE, intracellular ROS levels remained unchanged; however, treatment with HCIE at concentrations above 50 ng/mL significantly reduced these levels (Figures 4B,C).

### 3.5 HCIE inhibits MAPK activation in human keratinocytes

**FIGURE 5:**
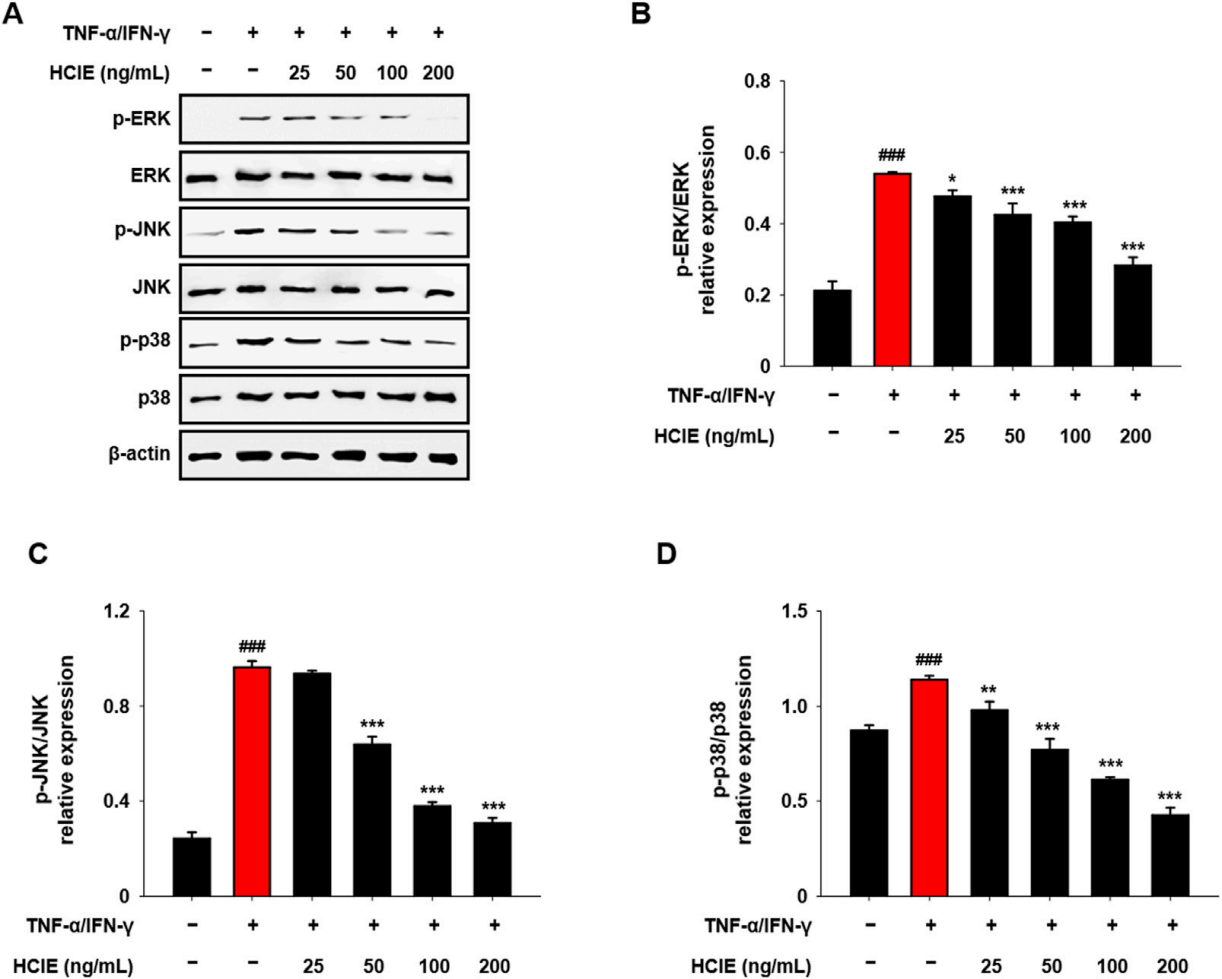
Reduced MAPK phosphorylation in HCIE-treated HaCaT cells. HaCaT cells were pre-treated with HCIE (25, 50, 100, and 200 ng/mL) for 30 min and then stimulated with 10 ng/mL of TNF-α/IFN-γ. The protein expression level of **(A)** MAPK was evaluated by immunoblotting. The phosphorylation levels of **(B)** ERK, **(C)** JNK, and **(D)** p38 were calculated as the ratio of the phosphorylated form to the total form. Data represented as mean ± SD (*n* = 3) and the *p* values are presented on the graph as follows: ^###^ *p* < 0.001 vs. control group; *p < 0.05, **p < 0.01, and ***p < 0.001 vs. TNF-α/IFN-γ induced group.

To investigate the effect of HCIE on skin inflammation, the phosphorylation of MAPK was assessed in HCIE-treated HaCaT cells. The phosphorylation of ERK, JNK, and p38 was decreased in a dose-dependent manner. In the group treated with 200 ng/mL of HCIE, the p-ERK/ERK ratio was 0.2827 ± 0.0264, the p-JNK/JNK ratio was 0.3076 ± 0.0229, and the p-p38/p38 ratio was 0.4268 ± 0.0392 (Figure 5).

### 3.6 HCIE regulates NF-κB translocation in human keratinocytes

**FIGURE 6:**
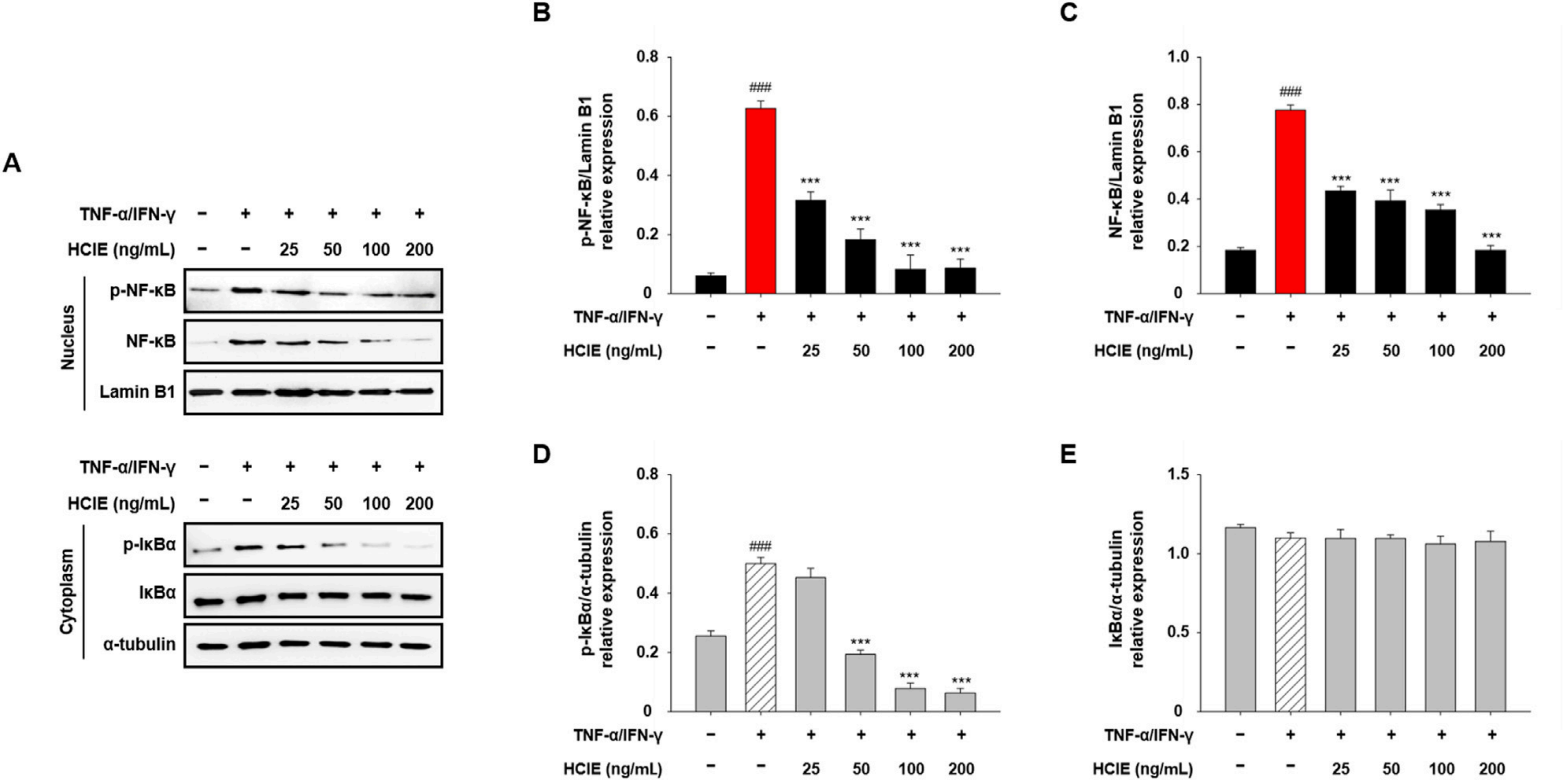
Inhibition of NF-κB translocation in HCIE-treated HaCaT cells. The cells were pre-treated with the indicated concentrations of HCIE for 30 min and stimulated with TNF-α/IFN-γ (each 10 ng/mL). Then, the cells were fractionated into the nucleus and cytoplasm. **(A)** Protein expression levels of NF-κB, p-NF-κB, IκBα, and p-IκBα were assessed by immunoblotting. Lamin B1 and α-tubulin were used as loading controls for the nucleus and cytoplasm, respectively. The relative expressions of **(B)** p-NF-κB, **(C)** NF-κB, **(D)** p-IκBα, and **(E)** IκBα were calculated as a ratio to the loading control. Data represented as mean ± SD (*n* = 3) and the *p* values are presented on the graph as follows: ^###^ *p* < 0.001 vs. control group; ***p < 0.001 vs. TNF-α/IFN-γ induced group.

Following HCIE treatment, the translocation of NF-κB to the nucleus was evaluated in HaCaT cells. NF-κB and p-NF-κB were decreased in the nucleus of HCIE-treated HaCaT cells, and p-IκBα was also reduced in the cytoplasm. NF-κB and p-NF-κB in the nucleus were significantly reduced even with 25 ng/mL of HCIE treatment, and p-IκBα in the cytoplasm was reduced in the group treated with 50 ng/mL or higher of HCIE (Figure 6).

### 3.7 HCIE inhibits the activation of NLRP3 inflammasome in human keratinocytes

**FIGURE 7:**
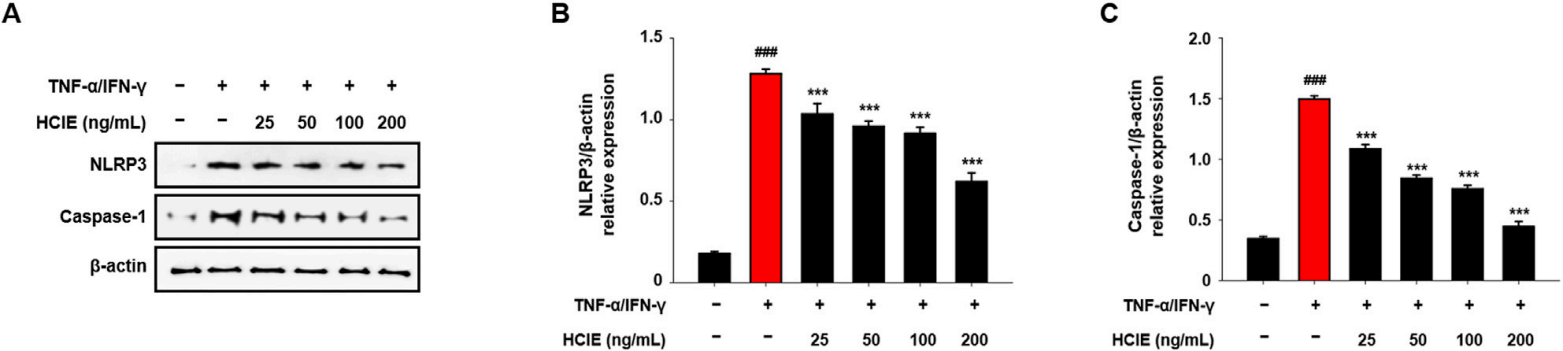
Inhibition of NLRP3 inflammasome activation in HCIE-treated HaCaT cells. HaCaT cells were pre-treated with HCIE (25, 50, 100, and 200 ng/mL) for 30 min and then stimulated with 10 ng/mL of TNF-α/IFN-γ. **(A)** The protein expression levels of NLRP3 and caspase-1 were evaluated by immunoblotting. The relative expressions of **(B)** NLRP3 and **(C)** caspase-1 were calculated as a ratio to the control. Data represented as mean ± SD (*n* = 3) and the *p* values are presented on the graph as follows: ^###^ *p* < 0.001 vs. control group; ***p < 0.001 vs. TNF-α/IFN-γ induced group.

To confirm the effect of HCIE on NLRP3 inflammasome, the expression levels of NLRP3 and caspase-1 were measured in HCIE-treated HaCaT cells. NLRP3 and caspase-1 were increased by TNF-α/IFN-γ stimulation and decreased by HCIE in a dose-dependent manner. The expression of NLRP3 was reduced by approximately 53% compared to the control group, while caspase-1 levels were decreased by approximately 68% (Figure 7).

### 3.8 HCIE regulates Th2 cytokines through inhibition of JAK1/STAT6 pathway

**FIGURE 8:**
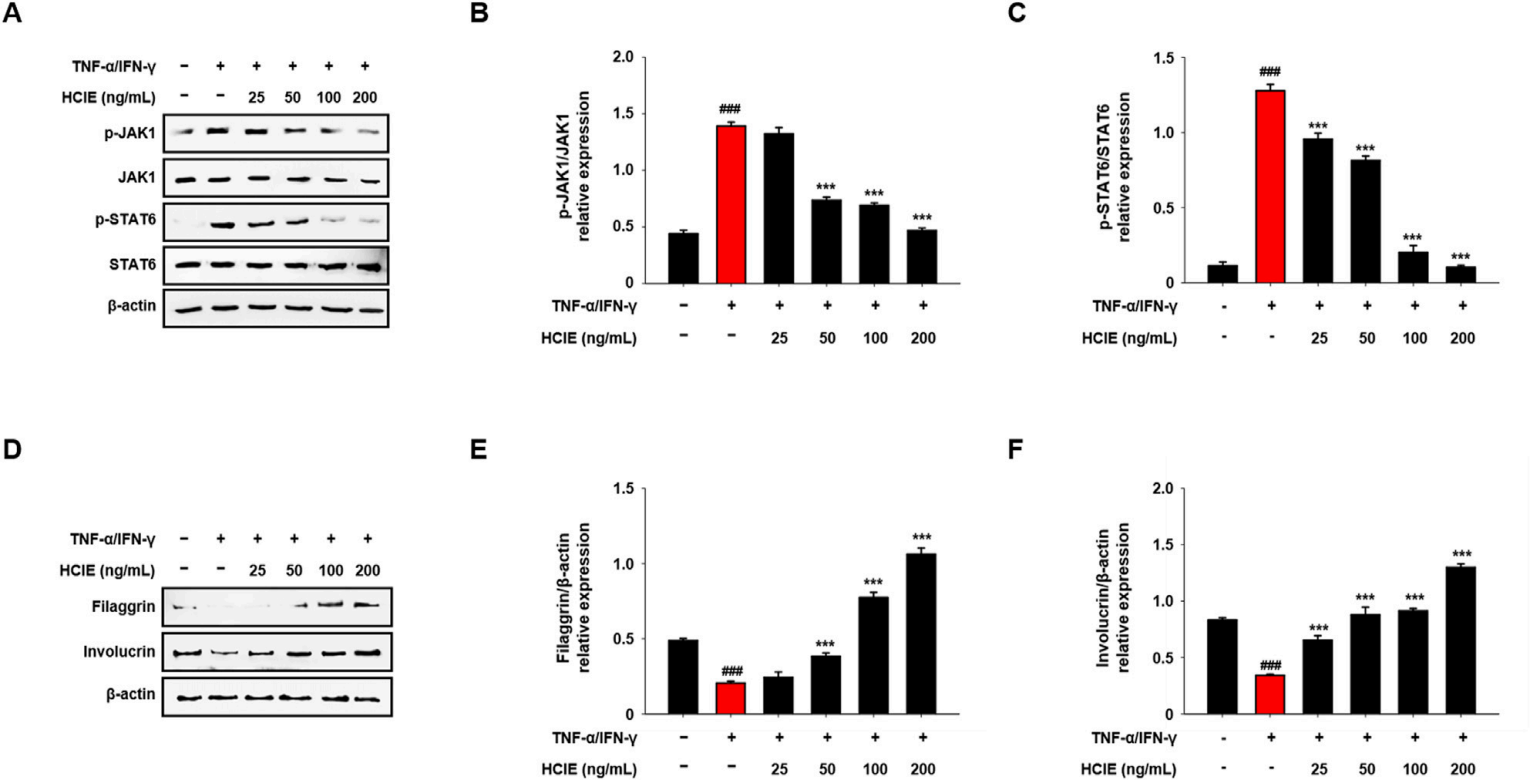
Regulation of JAK1/STAT6 activation and skin moisturizing factor in HCIE-treated HaCaT cells. The cells were pre-treated with the indicated concentrations of HCIE for 30 min and stimulated with TNF-α/IFN-γ. **(A)** The protein expression levels of JAK1 and STAT6 were evaluated by immunoblotting. The phosphorylation levels of **(B)** JAK1 and **(C)** STAT6 were calculated as the ratio of the phosphorylated form to the total form. **(D)** The protein expression levels of filaggrin and involucrin were evaluated by immunoblotting. The relative expressions of **(E)** filaggrin and **(F)** involucrin were calculated as a ratio to the control. Data represented as mean ± SD (*n* = 3) and the *p* values are presented on the graph as follows: ^###^ *p* < 0.001 vs. control group; ***p < 0.001 vs. TNF-α/IFN-γ induced group.

To investigate the effect of HCIE on Th2 response, the changes JAK1 and STAT6 were analyzed in HCIE-treated HaCaT cells. Phosphorylated JAK1 was significantly reduced in the HCIE treatment group above 50 ng/mL, and phosphorylated STAT6 was greatly reduced in the HCIE treatment group above 25 ng/mL. Phosphorylated JAK1 and STAT6 were most significantly reduced in the HCIE treatment group of 200 ng/mL. The expression ratios of phosphorylated JAK1 and STAT6 in the HCIE treatment group at a concentration of 200 ng/mL were 0.4681 ± 0.0218 and 0.1033 ± 0.0135, respectively (Figures 8A–C).

### 3.9 HCIE increases the skin moisturizing factor in human keratinocytes

To explore the effects of HCIE on skin moisture factors, the levels of intracellular filaggrin and involucrin were analyzed following HCIE treatment in HaCaT cells. The levels of intracellular filaggrin and involucrin were decreased upon TNF-α/IFN-γ stimulation but were subsequently restored following HCIE treatment. The levels of filaggrin were increased in the HCIE treatment group at concentrations above 100 ng/mL compared to the normal control group, with levels more than twice as high in the HCIE treatment group at 200 ng/mL relative to the normal control group. Furthermore, the levels of involucrin significantly increased in the HCIE treatment group at concentrations above 50 ng/mL (Figures 8D–F).

## 4 Discussion

AD is a common skin inflammatory disease, yet no ideal therapeutic agent or treatment has been established. In this study, the efficacy of a hybrid *C. sativa* developed in Korea to improve skin inflammatory diseases was evaluated, and its underlying mechanisms were investigated. The anti-inflammatory effects of HCIE were exerted through the modulation of key signaling pathways, including MAPK, NF-κB, and the NLRP3 inflammasome. In addition, HCIE promoted the expression of skin moisturizing factors in TNF-α/IFN-γ induced HaCaT cells.

Phytocannabinoids are compounds present in the cannabis plant, with CBD and THC constituting a significant proportion and being the most widely recognized (Walsh et al., 2021). The cannabinoids present in HCIE were analyzed using UPLC. Eight cannabinoids (CBDV, CBDVA, CBD, CBDA, CBGA, Δ^9^-THC, CBC, THCA) were detected in HCIE. Among these, the concentration of CBDA was the highest, with the content of CBD being approximately four times greater than that of THC. This suggests that hybrid *C. sativa* is a variety of hemp, which is non-psychoactive, in contrast to marijuana. According to previous studies, *C. sativa* exhibits diverse therapeutic effects depending on the relative content of THC and CBD. Varieties with high THC content have been primarily associated with analgesic effects, whereas those with high CBD content have demonstrated anticonvulsant and anti-inflammatory properties (Walsh et al., 2021). In particular, CBD exerts anti-inflammatory effects through multiple mechanisms, including inhibition of NF-κB signaling, down-regulation of proinflammatory cytokines activation, inhibition of oxidative stress, and modulation of immune cell activity (Martinez Naya et al., 2023). Similar to CBD, CBDA also exerts anti-inflammatory effects through the suppression of inflammatory cytokines and inhibition of cyclooxygenase-2 activity (Nigro et al., 2022). Based on this evidence, our findings suggest that CBD and CBDA, which are abundantly present in HCIE, are key compounds contributing to the improvement of AD.

AD is a chronic inflammatory skin disease. Pro-inflammatory cytokines/chemokines, such as IL-1β, IL-6, IL-8, and MCP-1, are closely associated with the upregulation of inflammation (Zhang and An, 2007). In particular, aberrant Th2 immune responses contribute to the pathogenesis of AD. In AD lesions, the expression of Th2 cytokines (IL-4 and IL-13) is markedly elevated, accompanied by the secretion of chemokines, including MDC, RANTES, TARC, and CXCL10 (Renert-Yuval et al., 2021). The gene expression of pro-inflammatory cytokines/chemokines was reduced by up to 82.2% in HCIE-treated HaCaT cells. Similarly, the gene expression of cytokines/chemokines associated with AD was significantly decreased in HCIE-treated HaCaT cells, with IL-4 being the most significantly reduced. Additionally, the secretion of RANTES and TARC was also diminished. This suggests that HCIE modulates the activity and migration of cells involved in inflammation, including mast cells, T cells, macrophages, neutrophils, and dendritic cells, by suppressing the gene expression of cytokines/chemokines as well as their extracellular secretion.

Intracellular ROS are unstable molecules that are naturally produced during cellular metabolism and induce inflammation (Liu et al., 2023). When they accumulate excessively, they cause oxidative stress, which contributes to the development of chronic inflammatory diseases (Chen et al., 2024). Intracellular ROS in keratinocytes affects the development of skin inflammatory diseases, such as AD (Okayama, 2005). The antioxidant effect of HCIE was demonstrated through DPPH radical scavenging activity and intracellular ROS levels measurement. The DPPH radical scavenging activity of HCIE increased in a dose-dependent manner, while the intracellular ROS level was significantly reduced in HCIE-treated HaCaT cells. These data suggest that HCIE exerts antioxidant effects and modulates inflammation by regulating intracellular oxidative stress, indicating that HCIE may contribute to the amelioration of inflammatory diseases.

Activation of MAPK and NF-κB pathways by Toll-like receptor signaling is a major mechanism of inflammation; the NF-κB pathway is closely linked to the NLRP3 inflammasome (Li et al., 2017). These pathways regulate the gene expression of cytokines/chemokines by modulating their transcription in the nucleus, ultimately regulating the activity of immune cells (Liu et al., 2017). In TNF-α/IFN-γ induced HaCaT cells, phosphorylation of MAPK and translocation of NF-κB were inhibited by HCIE. Similarly, Inhibition of NLRP3 inflammasome was also observed in HCIE-treated HaCaT cells. Given that MAPK and NF-κB pathways regulate the transcription of inflammatory mediators, and that NLRP3 inflammasome is involved in the activation of inflammatory cytokines, these findings suggest that HCIE modulates the transcription and activation of inflammatory mediators by controlling key mechanisms related to inflammation.

Activation of helper T cells and collapse of the skin barrier are key characteristics of AD. Th2 cytokines play a crucial role in the pathogenesis of AD. In AD lesions, the expression of Th2 cytokines, including IL-4 and IL-13, is upregulated, and they cause itching of the skin (Haddad et al., 2022). Th2 cytokines transmit signals by crossing the cell membrane via the JAK/STAT pathway, thereby modulating the immune response and promoting the development of AD (Huang et al., 2022). In addition, defects or loss of skin moisturizing factors, such as filaggrin and involucrin, are observed in AD lesions (Agrawal and Woodfolk, 2014). The collapse of the skin barrier causes moisture loss and changes in the skin’s pH, resulting in skin dryness and creating an environment that is more susceptible to infection (Rerknimitr et al., 2018). These altered skin conditions contribute to a vicious cycle of itch-scratch, which further worsens AD (Tominaga and Takamori, 2022). The inhibitory effect of HCIE on JAK/STAT activation was observed in HaCaT cells. Conversely, filaggrin and involucrin were increased by HCIE. These results imply that HCIE affects the onset and progression of AD through inhibition of JAK1/STAT6 phosphorylation and regulation of skin moisturizing factors.

Topical steroids and biological agents are used to treat atopic dermatitis (Davis et al., 2024). As previously mentioned, prolonged use of topical steroids can lead to significant side effects, including skin atrophy and adrenal dysfunction (Stacey and McEleney, 2021). JAK inhibitors, a class of biological agents, are used in the treatment of severe atopic dermatitis (Mikhaylov et al., 2023). However, the JAK/STAT pathway is critically involved in various physiological processes, including inflammation, immune response regulation, hematopoiesis, and cell proliferation. Therefore, the use of JAK inhibitors for inflammation suppression carries significant risks (Hu et al., 2021). Additionally, the use of JAK inhibitors should be approached with caution, as side effects such as increased susceptibility to viral and bacterial infections, joint disorders, and tumor formation have been reported (Hoisnard et al., 2022). The inhibition of JAK1/STAT6 activation by HCIE suggests its potential as a promising alternative to conventional JAK inhibitors in the treatment of AD.

This study demonstrated the antioxidant, anti-inflammation, and anti-atopic dermatitis effects of HCIE in an *in vitro* model. These effects were mediated through the regulation of the MAPK/NF-κB/NLRP3 inflammasome, JAK1/STAT6 pathway, and skin moisturizing factors. Nevertheless, the current *in vitro* data alone may not be sufficient to fully explain the symptom-relieving effects of HCIE in the context of AD. Therefore, further studies using *in vivo* models of AD are necessary to confirm the therapeutic efficacy of HCIE and to ensure its safety for clinical application.

Taken together, HCIE exhibited anti-atopic dermatitis effects in keratinocytes by down-regulating inflammatory mediators and up-regulating skin moisturizing factors. These results suggest that HCIE may serve as a beneficial agent for the treatment of skin inflammation and AD.

## 5 Conclusion

HCIE exerts anti-inflammatory and anti-atopic dermatitis effects in human keratinocytes by modulating the MAPK/NF-κB/NLRP3 inflammasome, JAK1/STAT6 pathway, and skin moisturizing factors. To further elucidate the effects of HCIE on AD, additional studies using *in vivo* models are required. Nevertheless, considering the effects of HCIE proven in this study, HCIE is a valuable substance for improving skin inflammation, potentially serving as an effective therapeutic agent or adjuvant for AD.

## Data Availability

The original contributions presented in the study are included in the article/Supplementary Material, further inquiries can be directed to the corresponding author.
