# Supplementary material for: Hybrid Cannabis sativa L. inflorescences exert an anti-inflammatory effect through the modulation of MAPK/NF-κB/NLRP3 inflammasome and JAK1/STAT6 pathway in HaCaT cells
Source: Front Pharmacol. 2025 Jul 24;16:1617180. doi: 10.3389/fphar.2025.1617180 (PMC12329069; doi:10.3389/fphar.2025.1617180)
Supplement: Supplementary file 1 [file Table1.docx]

Table S1. Information on cannabinoid standards

| **Type** | **Cannabinoid** | **Concentration** | **Manufacturer**  **(Cat No.)** |
| --- | --- | --- | --- |
| Single | CBC | 1 mg/mL | Absolute standards (73604) |
|  | CBL | 1 mg/mL | Cayman (22036) |
|  | CBDA | 1 mg/mL | Absolute standards (73683) |
|  | CBDV | 1 mg/mL | Absolute standards (74129) |
|  | CBGA | 1 mg/mL | Absolute standards (73683) |
|  | CBNA | 1 mg/mL | Cayman (21734) |
|  | CBDVA | 1 mg/mL | Cayman (30245) |
|  | THCVA-A | 1 mg/mL | Cayman (21259) |
|  | Δ8-THC | 1 mg/mL | Absolute standards (79879) |
|  | CBG | 1 mg | Cayman (15293) |
| Cannabinoids  Mixture | Δ9-THC | 0.075 mg/mL | U.S Pharmacopeia (1089183) |
|  | Δ8-THC | 0.025 mg/mL |  |
|  | CBD | 0.050 mg/mL |  |
|  | CBN | 0.025 mg/mL |  |
|  | CBC | 0.025 mg/mL |  |
|  | CBG | 0.025 mg/mL |  |
|  | THCV | 0.025 mg/mL |  |
|  | CBDV | 0.025 mg/mL |  |
| Cannabinoid  Acids Mixture | THCA | 0.025 mg/mL | U.S Pharmacopeia (1089172) |
|  | CBDA | 0.025 mg/mL |  |
|  | THCVA | 0.050 mg/mL |  |
|  | CBDVA | 0.025 mg/mL |  |
|  | CBGA | 0.025 mg/mL |  |

Table S2. Primer list for Real-time PCR

| **Gene** |  | **Primer sequence** |
| --- | --- | --- |
| *IL-1β* | Forward: | 5’-CTCTCTCACCTCTCCTACTCAC-3’ |
|  | Reverse: | 5’-ACACTGCCTACTTCTTGCCCC-3’ |
| *IL-4* | Forward: | 5’-ACATTGTCACTGCAAATCGACACC-3’ |
|  | Reverse: | 5’-TGTCTGTTACGGTCAACTCGGTGC-3’ |
| *IL-6* | Forward: | 5’-CTCCAC AAGCGCCTTCGGTC -3’ |
|  | Reverse: | 5’-TGTGTGGGGCGGCTACATCT-3’ |
| *IL-8* | Forward: | 5’-ACCGGAGCACTCCATAAGGCA-3’ |
|  | Reverse: | 5’-AGGCTGCCAAGAGAGCCACG-3’ |
| *IL-13* | Forward: | 5’-ACCACGGTCATTGCTCTCACT-3’ |
|  | Reverse: | 5’-GTCAGGTTGATGCTCCATAC-3’ |
| *MCP-1* | Forward: | 5’-TCTGTGCCTGCTGCTCATAG-3’ |
|  | Reverse: | 5’- CAGATCTCCTTGGCCACAAT-3’ |
| *CXCL10* | Forward: | 5’-TTGCTGCCTTATCTTTCTGACTC-3’ |
|  | Reverse: | 5’-ATGGCCTTCGATTCTGGATT-3’ |
| *RANTES* | Forward: | 5’-CGCTGTCATCCTCATTGCTA-3’ |
|  | Reverse: | 5’-GCACTTGCCACTGGTGTAGA-3’ |
| *TARC* | Forward: | 5’-CCATTCCCCTTAGAAAGCTG-3’ |
|  | Reverse: | 5’-CTCTCAAGGCTTTGCAGGTA-3’ |
| *MDC* | Forward: | 5’-TGCCGTGATTACGTCCGTTAC-3’ |
|  | Reverse: | 5’-AAGGCCACGGTCATCAGAGTAG-3’ |
| *GAPDH* | Forward: | 5’-GAAGGTGAAGGTCGGAGT-3’ |
|  | Reverse: | 5’-GAAGATGGTGATGGGATTTC-3’ |

Table S3. Information on antibodies used for immunoblotting

| **Antibody** | **Manufacturer** | **Cat No.** | **Dilution ratio** |
| --- | --- | --- | --- |
| p-ERK | Cell Signaling Technology | #9101 | 1:1000 |
| ERK | Cell Signaling Technology | #4695 | 1:1000 |
| p-JNK | Cell Signaling Technology | #4668 | 1:1000 |
| JNK | Cell Signaling Technology | #9252 | 1:1000 |
| p-p38 | Cell Signaling Technology | #4511 | 1:1000 |
| p38 | Cell Signaling Technology | #9212 | 1:1000 |
| p-NF-κB | Cell Signaling Technology | #3033 | 1:1000 |
| NF-κB | Cell Signaling Technology | #8242 | 1:1000 |
| p-IκBα | Cell Signaling Technology | #2859 | 1:1000 |
| IκBα | Cell Signaling Technology | #9242 | 1:1000 |
| Lamin B1 | Cell Signaling Technology | #13435 | 1:1000 |
| α-tubulin | Cell Signaling Technology | #2144 | 1:1000 |
| NLRP3 | Cell Signaling Technology | #15101 | 1:500 |
| Caspase-1 | Santa Cruz Biotechnology | sc-56036 | 1:1000 |
| p-JAK1 | Cell Signaling Technology | #3331 | 1:1000 |
| JAK1 | Cell Signaling Technology | #3344 | 1:1000 |
| p-STAT6 | Cell Signaling Technology | #56554 | 1:1000 |
| STAT6 | Cell Signaling Technology | #5397 | 1:1000 |
| Filaggrin | Santa Cruz Biotechnology | sc-66192 | 1:500 |
| Involucrin | Santa Cruz Biotechnology | sc-21748 | 1:1000 |
| β-actin | Cell Signaling Technology | #4970 | 1:1000 |
